# Measuring the neglected anxiety disorder: validation of the social anxiety spectrum-short version (SHY-SV) questionnaire

**DOI:** 10.1186/s12888-023-05137-5

**Published:** 2023-10-02

**Authors:** Liliana Dell’Osso, Ivan Mirko Cremone, Benedetta Nardi, Giulia Amatori, Chiara Bonelli, Davide Gravina, Francesca Benedetti, Luca Del Prete, Gabriele Massimetti, Barbara Carpita

**Affiliations:** Department of Clinical and Experimental Medicine, University of Pisa, Via Roma 67, Pisa, 56127 Italy

**Keywords:** Social anxiety spectrum, SAD, Spectrum model, Questionnaire

## Abstract

**Background:**

In the recent years, a growing body of literature stressed the importance of a dimensional perspective on mental disorders. In particular, since its conceptualization, one of the main concerns in the field of Social Anxiety Disorder (SAD) has been the definition of a diagnostic threshold, leading to the suggestion that SAD may be more properly classified as a spectrum of severity rather than a discrete disorder based on subjectively determined threshold. The purpose of the current research is to evaluate the psychometric qualities of the Social Anxiety Spectrum - Short Version (SHY-SV), a novel questionnaire designed to measure the complete range of social anxiety symptoms, from overt manifestations to subthreshold ones.

**Methods:**

42 subjects with a clinical diagnosis of social anxiety disorder (SAD) according to the Diagnostic and Statistical Manual of Mental Disorders (DSM-5), 43 subjects with a clinical diagnosis of Obsessive-Compulsive Disorder (OCD) and 60 individuals without current or lifetime mental disorders (HC) were recruited from the Psychiatric Clinic of the University of Pisa. Subjects were assessed with the SCID-5, Liebowitz Social Anxiety Scale (LSAS) and the SHY-SV.

**Results:**

SHY-SV showed strong internal consistency, and both the total and domain scores had great test-retest reliability. The Pearson’s coefficients for the SHY-SV domain scores ranged from 0.391 to 0.933, and they were positively and significantly correlated with one another (p 0.001). All the SHY-SV domain scores were highly correlated with the SHY-SV total score. Results from of the correlation coefficients between SHY-SV and alternative measures of SAD were all significant and positive. Significant differences among diagnostic groups on both SAD-SV domains and total scores were found. SAD-SV total score increased significantly and progressively from HCs, to the OCD up to the SAD group which showed the highest values.

**Conclusion:**

The SHY-SV demonstrated significant convergent validity with other dimensional SAD measures, great internal consistency, and test-retest reliability. With an increasing score gradient from healthy controls to patients with OCD to those with SAD, the questionnaire performed differently in each of the three diagnostic categories.

## Background

The Social Anxiety Disorder (SAD) was firstly described by Pierre Janet [1] in the beginning of 20th century under the name of Social Phobia (SP). First grouped among specific phobias [2], SAD gained diagnostic dignity in the third edition of the Diagnostic and Statistical Manual of Mental Disorders (DSM) [3], and only in the fourth the name was changed in its current one, which better highlights the impairment and pervasiveness of the condition, firmly differentiating it from specific phobias [4, 5]. SAD in characterized by persistent fear of one or more social situations or performances in which the person is exposed to non-familiar people or to a potential judgment by others that, as a result, lead the individual to avoid the feared situation or face it with excruciating anxiety or distress [6]. Despite wanting to be with others, subjects with SAD tend to refrain from social situations and avoid expressing their opinions out of concern that they would be viewed as unreliable or stupid [7]. The lifetime prevalence estimated in the general population varies from 1.9 to 13.7% [8, 9]. Typically, SAD symptoms begin early in life, even in childhood [7], persisting through the entire school career [10] and leading to negative effects on the academic performances such as school interruption, increased possibility of failing exams [11] and lack of graduation [12, 13]. SAD frequently coexists with other mental disorders, in particular with mood disorders such as major depression (MDD), dysthymia, or bipolar disorder (BD) [14, 15], as well as with other anxiety disorders like obsessive compulsive disorder (OCD) [16], generalized anxiety disorder (GAD) [17], panic disorder (PD) [18], body dysmorphic disorder (BDD) [19], or even substance abuse. In particular, many studies highlighted the high comorbidity between social anxiety and alcohol/cannabis use disorder [20, 21] and even how subjects with subclinical traits of social anxiety (SA) have a greater risk of incur in an alcohol or cannabis use disorder than the non-clinical population [22, 23]. Moreover, people with SAD may be more susceptible to problematic substance use in order to avoid being negatively judged by others [22], to come with internal distress or to conform and fit in with their peers [24–26].

Although being quite neglected in clinical settings, to the point of gaining the label of ‘‘the neglected anxiety disorder’’ [27], SAD is a rather frequent and impairing condition that in the past years raised interest in many researchers [28, 29]. Noticeably, SAD has been reported to be frequently under-recognized, due to the same nature of the disorder, which increases the tendency to avoid contacts with other subjects, including clinicians, but also due to socio-cultural factors and prejudices about the acceptability of shyness, especially among women [30, 31].

Since its conceptualization, one of the main concerns in the field of SAD was the definition of a diagnostic threshold [32]. Many researchers suggested that SAD may be more properly classified as a spectrum of severity rather than a discrete disorder based on subjectively determined threshold [33] and that boundaries of SAD should be determined by its severity rather than by qualitative characteristics [4, 34, 35]. In line with this view, the latest editions of the DSM [6, 36] apported some changes in the chapter dedicated to SAD, reflecting a new and greater understanding of such condition in various social situations. In particular, while in previous descriptions of the disorder the presence of acute discomfort or dread when performing in front of other people used to be the primary criterion for the diagnosis of SAD [37], the new editions of the manual removed the distinction between the generalized and specific forms, increasing the range of circumstances in which a person may have social anxiety symptoms and removed the requirement of being aged over 18 years and of recognizing the excessiveness and unreasonableness of their discomfort, prompting a re-consideration of symptoms distribution in non-clinical populations [28]. A specifier was instead added for the subtype “performance only”, which should be used when SAD symptoms only arise when the subjects have to speak or do other performance in front of an audience. Interestingly, a major change revolved around the objectivity of the disproportionality of anxiety symptoms: this therefore allows including in the clinical evaluation even individuals who judge their reaction to be normal in certain situations due to their belief of having a “constitutional shyness” or due to the lack of complete awareness of their symptoms. Following this conceptualization, several studies hypothesized that SAD would be better categorized as dimensional continuum [33].

According to such literature, the wide sub-threshold manifestations that may coexist with the major mental disorder can be more easily identified using a spectrum model of psychopathology [38]. In this context, the term “spectrum” in used to describe mental health conditions that cover a range of symptoms and behavioral traits connected to a recognized DSM or ICD illness construct (like depression, panic or obsessive compulsive disorders) [39]. While the primary symptoms of the current DSM diagnostic categories are included in the spectrum of symptoms and traits, the spectrum conceptualization also includes sub-clinical and atypical manifestations, in addition to temperamental and/or personality traits and isolated signs and symptoms, symptom clusters, and behavioral patterns [32, 39–46]. In this view, the spectrum symptomatology can be compared to the part of an iceberg that is hidden under water surface, whereas the full-blown diagnostic criteria symptoms represent the visible portion [39].

According to this model, in the early 2000’s, the “Social Phobia Spectrum Self-report” (SHY-SR) instrument was developed and validated in the context of the “spectrum project”, an international collaboration with the purpose of share light about the validity of a dimensional approach to psychopathology [32, 38–46].

The SHY-SR aims to assess not only the prototypic symptoms of SAD but also atypical manifestations, temperamental traits, and other noteworthy clinical and sub-clinical aspects linked to the main symptoms [41, 42]. The questionnaire demonstrated high internal consistency, and a good inter-rater reliability along with good discriminant validity. During the last decades, it was used in different clinical settings [30, 31, 47, 48]. However, due to the extended time needed to complete it – up to 60 min – its implementation in regular clinical practice has remained quite challenging. Additionally, the instrument, being tailored on DSM-IV TR criteria, still included outdated and unnecessary components.

As the main authors of the SHY-SR, we aimed to develop a new revised and shortened version of the questionnaire, the Social Anxiety Spectrum– Short Version (SHY-SV) which should report a shorter compilation time as well as higher internal consistency, inter-rater reliability, and discriminant validity. The new instrument was developed including more contemporary items and excluding older ones, in order to be a more useful and update tool for clinical practice and research on both the full-blown and milder subsyndromal form of SAD. In this framework, the present work aimed to validate the SHY-SV questionnaire in a clinical population of patients with SAD patients, OCD patients and in healthy controls (HC). In particular, considering the reported presence of sub-threshold SAD traits in subjects with OCD and the overlapping features between SAD and OCD spectra, the OCD group was recruited as a potential intermediate group for SAD traits between SAD patients and HC [48].

## Methods

Data have been collected between September 2022 and December 2022 at the Psychiatric clinic of the University of Pisa.

### Study sample and procedure

The total sample consisted in 145 subjects distributed in three diagnostic groups, all evaluated according to DSM-5 diagnostic criteria. The SAD and the OCD subjects were recruited from out- patients afferent at the Psychiatric Clinic of the University of Pisa. The recruitment of HC and OCD samples was carried following a sex- and gender-matched criteria. Exclusion criteria were age below 18 years, language or intellectual impairment affecting the possibility to fill out the assessments, mental disability, poor cooperation skills and ongoing psychotic symptoms. Specifically, the four groups were individuated as follows: 42 subjects with a clinical diagnosis of SAD; 43 subjects with a clinical diagnosis of OCD and 60 individuals without current or lifetime mental disorders (HC) belonging to health care and paramedical personnel. All subjects in order to be recruited must be aged between 18 and 70 and willing to sign an informed consent. The Structured Clinical Interview for DSM-5, Research Version (SCID-5-RV) [49] was used to confirm the diagnosis of SAD and OCD, as well as the absence of mental disorders among HC subjects. Subjects belonging to the clinical sample were not diagnosed with BD or major depressive disorder, however, a depressive episode was contemplated in a minority of subjects as long as the depressive symptoms were less prominent than those of the category disorder. Similarly, the presence of other anxiety disorders was accepted as long as the symptoms were significantly less prominent than those of the OCD or SAD. The test-retest reliability of the SHY-SV, performed in order to provide evidence for the temporal stability of the scores, was determined on 30 subjects randomly extracted from study sites and by means of a second evaluation over an interval of 21 days from the initial assessment. In the test-retest group, no changes in drug therapy were made during the time between the first and second evaluation. The study was conducted in accordance with the Declaration of Helsinki. Eligible subjects provided written informed consent, after receiving a complete description of the study and having the opportunity to ask questions. Subjects were not paid for their participation.

### Measures

Assessment procedures included the SCID-5-RV [49], the Liebowitz Social Anxiety Scale (LSAS), and the Social Anxiety Spectrum - Short Version questionnaire (SHY-SV). Questionnaire evaluations were carried by psychiatrists, who were trained and certified in the use of the study instruments.

#### The liebowitz social anxiety scale (LSAS)

The LSAS is one of the most widely utilized scales to clinically assess the severity of social anxiety symptoms in a range of social interactions and performances [50]. The LSAS was originally conceptualized as a clinician-administered rating scale, but has later been validated as a self-report scale [51]. The scale features 24 items divided in 2 subscales: 13 items focus on performance anxiety and 11 concerns social situations. The 24 items are rated on a Likert scale ranging from 0 to 3 (none, mild, moderate, severe) and are firstly scored based on the fear felt during the situation and then based on the avoidance of the same situations. The overall total score ranges from 0 to 144, while the scores for fear and avoidance subsections ranges from 0 to 72 [52]. The scale proved to have excellent validity, reliability and sensitivity [53].

#### The social anxiety spectrum - short version questionnaire (SHY-SV)

The SHY-SV consists in 139 items organized in 5 domains (108 items) and an appendix (8 items). The answers to the various items are coded in a dichotomous way (yes/no) and the scores relating to the single domains and appendices are obtained by counting the number of positive answers. The *Interpersonal sensitivity* domain (22 items) explore the issues linked to hypersensitivity to criticism, rejection and scrutiny, of the discomfort at being the focus of the attention, poor self- esteem as well as feeling of inferiority and difficulties in interpersonal relationship. The *Behavioral inhibition* (12 items) domain investigates peculiar behaviors or modification in then compared to the usual, as well as physical symptoms and some of the biological markers such as the tone of the voice, the ability to hold gaze, the posture and restlessness of the hands during social interactions. The *Performance* domain and the *Social situations* domain both investigate 6 areas, respectively through 30 and 40 items regarding performances and social situations related to social anxiety, anticipatory anxiety and avoidance. The *Substance use* domain concerns the use of psychoactive substances, for it is a quite common complication of SAD.

The appendix *Childhood and adolescence* contains 8 items and refers to social anxiety traits that may have emerged during childhood or adolescence in particular fear and/or avoidance of social activities and somatic symptoms that manifested at school, during the free time and in during the practice of sports.

Five trained clinicians (LDO, BC, BN, DG, BC) screened the items for inclusion and disagreements were resolved by discussion. The selection of the items relied upon the affinity with the clinical description of SAD provided by the DMS and the recent literature; items deemed dated, not applicable to the general population due to cultural or historical factors, ambiguous or easily misunderstood or non-discriminatory for the SAD spectrum were excluded. Of the 168 items present in the previous version, after a process of clinical selection, only 108 items were selected. Compared to the previous version, the *Interpersonal sensitivity* domain went from 29 to 22 items; the *Inhibited behavior* domain went from 23 to 12 items; the *Performance* domain went from 38 to 30 items; the *Social situation* domain went from 60 to 40 items, the *Substance use* domain went from 6 to 4 items. The *Childhood and adolescence* domain was transformed into an appendix and reduced from 12 to 8 items.

### Statistical analyses

The Cronbach’s alpha was determined for each domain and the questionnaire’s overall score to estimate the SHY-internal SV’s consistency. To ascertain how each item affected the instrument’s dependability, the changes in alpha with deleted items were evaluated. Computing bivariate Pearson’s correlation coefficients between the five domain scores and between each domain score and the overall score allowed researchers to examine the validity of the instrument’s internal structure. By calculating the intra-class correlation coefficient (ICC) on a subgroup of 30 participants randomly selected from the original database and re-evaluated after a gap of 3 weeks, the test-retest reliability of the questionnaire was examined. By measuring the Pearson’s correlation coefficients between the SHY-SV domain and total scores as well as the LSAS total score as a substitute for the SAD, the convergent validity was examined. The mean total and domain scores recorded in the three diagnostic groups were compared by a One-way analysis of variance to examine the instrument’s discriminatory ability (Known-groups validity) (ANOVA). Post-hoc comparisons were made using the Bonferroni Test. All statistical analyses were performed with SPSS version 26.0 [54].

## Results

The sociodemographic characteristic of the sample, including gender composition, mean age, educational level, occupational role and marital status and the corresponding table are reported elsewhere [55].

### Internal consistency and test-retest reliability

The Cronbach alphas and ICCs for the individual domains and the total score calculated for the entire sample are displayed in Table 1. A high level of internal consistency was shown by the SHY-SV scale. With the exception of the domain for substance use, all of the SHY domains’ Cronbach alpha values were good (exceeding the value of 0.8), and the value for the scale’s overall score is excellent (α = 0.975). The fact that the alpha value decreased as each item was eliminated shows that each one made a meaningful contribution to the scale. With all ICCs above the value of 0.90, the test-retest reliability for total and domain scores was outstanding.

**Table 1:** SHY-SV internal consistency and test-retest reliability

*SHY-SV domains*	*Number of items*	*Cronbach’s alpha*	*ICC*
*Interpersonal sensitivity*	22	0.940	0.986
*Behavioral inhibition*	12	0.876	0.974
*Performance*	30	0.924	0.987
*Social situations*	40	0.941	0.997
*Substance use*	4	0.692	0.921
**Total score**	108	0.975	0.997

### Validity of the internal structure

The Pearson’s coefficients for the SHY-SV domain scores ranged from 0.391 to 0.933, with the lowest value corresponding to the *Substance use* domain. These correlations were strong, positive and significant (p.001) for each domain, with the exception of the *Substance use* domain for which the correlation was moderate (0.391). The SHY total score and each of the SHY-SV domain scores had a positive correlation (see Table 2).

**Table 2:** Correlations among the SHY-SV domains^a^

*SHY Spectrum domains*	*Interpersonal sensitivity*	*Behavioral inhibition*	*Performance*	*Social situations*	*Substance use*
*Interpersonal sensitivity*	-	-	-	-	-
*Behavioral inhibition*	^0.770^	-	-	-	-
*Social situations*	^0.736^	^0.627^	-	-	
*Substance use*	^0. 447^	^0.391^	^0.498^	-	-
*Performance*	^0.671^	^0.555^	^0.794^	^0.439^	-
**Total score**	^**0.890**^	^**0.778**^	^**0.933**^	^**0.544**^	^**0.882**^

^*a*^ *Pearson’s correlation coefficients were all significant at the p < .01 level, two tailed*

### Convergent validity

The correlations between the LSAS *Fear* and *Avoidance* subscales and total score and the SHY-SV total and domain scores is shown in Table 3 via using Pearson’s correlation coefficients. All the correlation coefficients appeared strong, statistically significant and positive.

**Table 3:** Correlations between the SHY-SV domains and LSAS *Fear* and *Avoidance* subscales and total score ^a^

*SHY-SV domains*	*Fear*	*Avoidance*	*LSAS total score*
*Interpersonal sensitivity*	0.735	0.705	0.736
*Behavioral inhibition*	0.757	0.746	0.768
*Social situations*	0.751	0.667	0.725
*Substance use*	0.374	0.287	0.339
*Performance*	0.604	0.509	0.570
**Total score**	**0.795**	**0.721**	**0.776**

^*a*^ *Pearson’s correlation coefficients were all significant at the p < .01 level, two tailed*

### Known-groups validity

**Fig. 1:**
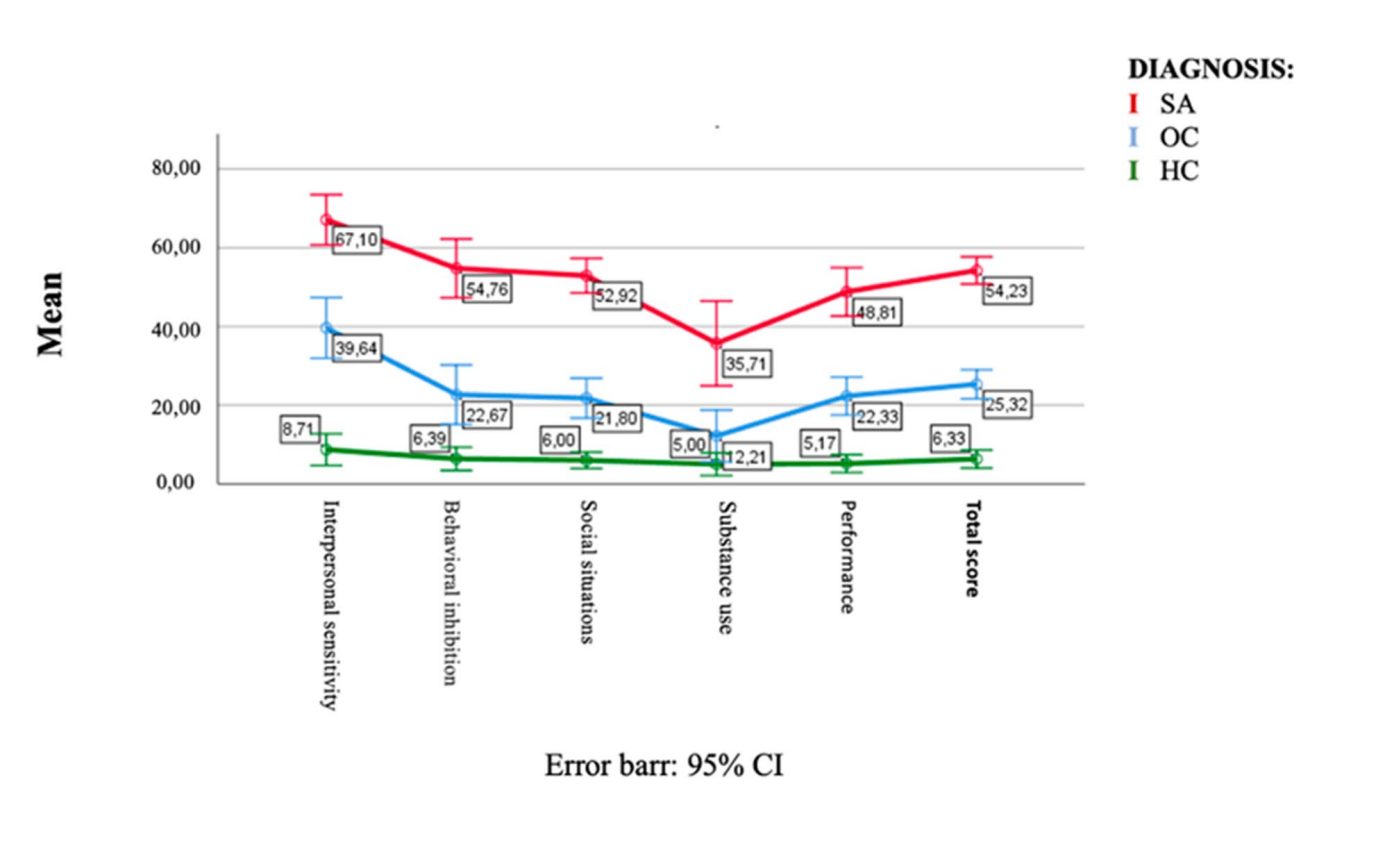
SHY-SV graph of normalized variables

The ANOVA analysis revealed significant variations between diagnostic groups on all SHY-SV domains and overall scores (see Table 4). Specifically, the SAD group scored significantly higher in all domains than the OCD group, which in turn scored significantly higher than the HC group in all domains, with the exception of the *Substance use* domain. Figure 1 illustrates the increasing trend of the SHY domain scores across groups, through a representation of the standardized mean scores of each domain and the total score, with SAD patients showing significantly higher scores than the other two groups.

**Table 4:** Comparison of SHY-SV total and domain scores among diagnostic groups

*SHY domains*	*HC* *(mean ± SD)*	*OC* *(mean ± SD)*	*SA* *(mean ± SD)*	*F*	*p*	*Post-hoc comparison* ^*a*^
*Interpersonal sensitivity*	1.92 ± 3.42	8.72 ± 5.51	14.76 ± 4.51	105.1	< 0.001	HC < OC < SA
*Behavioral inhibition*	0.77 ± 1.37	2.72 ± 2.94	6.57 ± 2.87	73.32	< 0.001	HC < OC < SA
*Social situations*	2.40 ± 3.19	8.72 ± 6.56	21.17 ± 5.64	167.35	< 0.001	HC < OC < SA
*Substance use*	0.20 ± 0.44	0.49 ± 0.85	1.43 ± 1.38	22.79	< 0.001	HC < SA OC < SA
*Performance*	1.55 ± 2.62	6.70 ± 4.69	14.64 ± 5.92	108.85	< 0.001	HC < OC < SA
**Total score**	**6.83 ± 9.62**	**27.35 ± 12.92**	**58.57 ± 11.96**	**256.12**	**< 0.001**	HC < OC < SA

^*a*^ *p < .001*

## Discussion

The purpose of this work was to present and measure the validity and reliability of the SHY-SV, a clinical instrument prompted by a dimensional approach to psychopathology, in light of the spectrum model [32, 40–46]. The SHY-SV assesses the core symptoms of SAD as well as the atypical manifestations, the temperamental traits and other remarkable clinical aspects associated with the central symptoms. The results of the study provided strong evidence of the validity and reliability of the SHY-SV, which was administered to a sample of subjects with a clinical diagnosis of SAD, OCD and individuals without current or lifetime mental disorders. We found excellent internal consistency and test-retest reliability and a significant and positive convergent validity with the alternative dimensional measures of SAD.

The questionnaire performed differently in each of the three groups studied, and the SHY-SV scores increased gradually from HC to OCD subjects up to SAD patients. The SHY-SV scores showed significant and strong positive correlations with the LSAS, one of the most popular tools used today to evaluate SAD symptoms and features [56–58]. Moreover, it is noteworthy to mention that the SHY-SV questionnaire appeared to be an instrument capable of identifying even subthreshold SAD traits in the OCD group and the non-clinical population, showing an increasing gradient of social anxiety traits from the HC, passing through OCD subjects up to the SAD group (Fig. 1). The presence of social anxiety traits at intermediate levels in the OCD population is consistent with previous research in the field, which frequently noted multiple social anxiety symptoms among OCD patients as well as similarities between the two disorders, further supporting a spectrum model of psychopathology [28, 30, 48, 59, 60]. Overall, our results support the capacity of the SHY-SV to accurately detect the whole spectrum of SAD, from the subthreshold manifestations to the full-blown clinical picture. However, some limitations concerning the study should be considered. The main limitation is the relatively small sample size, which might make our data less extensible. Furthermore, the SHY-SV, as the LSAS, are self-reported questionnaires, and subsequently may underestimate or overestimate symptoms based on the subjects’ perceptions, being less accurate than a clinician’s assessment. Interestingly, the substance abuse subscale shows several low correlations. Although the association of SAD with the use of alcohol and substances has been frequently reported in the literature, it is conceivable that, due to the nature of the SHY-SV a self-report questionnaire, has occurred a significant underestimation of the latter. This could also be explained by the fact that many subjects may not consider their use of substance as problematic. Moreover, even though in the test-retest group, no changes in drug therapy were made during the time between the first and second evaluation, specific information regarding the psychopharmacological therapy of the clinical subjects were not collected, excluding from the evaluation the possible inference of a psychopharmacological therapy. Lastly, the sample was not assessed with a measurement for depression nor for trait/state anxiety. In the context of those limitations, however, the SHY-SV demonstrated good psychometric properties and our results provide a coherent construct of the SHY-SV with strong internal consistency, high test-retest reliability and significative and positive convergent validity with alternative dimensional measures of SAD such as the LSAS. The SHY-SV has the advantage of being more time- and money-efficient and in line with the most recent descriptions of SAD when compared to the previous versions of the instrument and with face-to-face interviews [6, 36]. In this context, it should be noted that, in addition to OCD subjects, SAD traits have also been linked to a wide range of psychiatric disorders, including neurodevelopmental disorders, mood disorders, eating and feeding disorders, and personality disorders, frequently worsening the clinical picture and impacting treatment outcomes [14–19].

The availability of a tool that can identify sub-syndromic and atypical manifestations of this condition, which remain widely under-recognized, may improve diagnostic evaluation and treatment plans for the patients as well as support preventive and screening strategies in the general population. However, although the questionnaire demonstrated a good discriminating ability between the diagnostic categories and a good agreement with the diagnosis made by the clinician according to the DSM-5-TR criteria and through the SCID-5 diagnostic interview, the questionnaire alone is not sufficient for the diagnosis and should not be indicated as an alternative to the clinical interview, but rather as a supporting tool exploring the SA dimension in a dimensional way.

## Conclusion

The SHY-SV demonstrated significant convergent validity with other dimensional SAD measures, great internal consistency, and test-retest reliability. With an increasing score gradient from healthy controls to patients with OCD to those with SAD, the questionnaire performed differently in each of the three diagnostic categories.

## Data Availability

The raw data supporting the conclusions of this article will be made available by the authors upon request to prof. Liliana Dell’Osso (liliana.dellosso@unipi.it).

## Abbreviations

SAD: Social Anxiety Disorder
SP: Social Phobia
DSM: Diagnostic and Statistical Manual of Mental Disorders
MDD: Major depression
BD: Bipolar disorder
OCD: Obsessive compulsive disorder
PD: Panic disorder
BDD: Body dysmorphic disorder
SA: Social anxiety
SHY-SR: Social Phobia Spectrum Self-report
SHY-SV: Social Anxiety Spectrum– Short Version
HC: Healthy controls
LSAS: Liebowitz Social Anxiety Scale
ICC: Intra-class correlation coefficient
